# Evaluating the Diagnostic Accuracy and Challenges of the Two-Week Wait Referral Pathway for Skin Cancers in Primary Care

**DOI:** 10.7759/cureus.77410

**Published:** 2025-01-14

**Authors:** Harish Shivakumar, Upamanyu Leo Chanda, Ogba Onwuchekwa

**Affiliations:** 1 Internal Medicine, York and Scarborough Teaching Hospitals NHS Foundation Trust, York, GBR; 2 Ophthalmology, New Cross Hospital, Royal Wolverhampton Trust, Wolverhampton, GBR; 3 Consultation-Liaison Psychiatry, York and Scarborough Teaching Hospitals NHS Foundation Trust, York, GBR

**Keywords:** audit, dermatology, quality improvement, referral pathway, skin cancer

## Abstract

Skin cancers are among the most common cancers in the Western world, with incidence rates increasing significantly over time. Skin cancer survival rates are highly dependent upon early identification. In the United Kingdom (UK), initial assessment of skin lesions is carried out via general practitioners (GPs) who identify and refer suspected cases under the two-week pathway in compliance with the National Institute for Health and Care Excellence (NICE) guidelines. A major challenge in this pathway is the relatively low proportion of these referrals resulting in a skin cancer diagnosis. This retrospective study, conducted at a general practice encompassing 17,000 patients, evaluated the efficacy of primary care referral pathways for suspected skin malignancies and examined the key factors affecting diagnostic accuracy.

The *SystmOne* patient database was used to identify referral letters for suspected skin cancers, including squamous cell carcinoma, basal cell carcinoma, and melanoma, in a period between September 8th, 2021, and July 28th, 2022. A total of 146 referral letters were reviewed, with 35 selected for further analysis. The study highlighted that only 13% of referrals resulted in a confirmed diagnosis of skin cancer, falling below the local audit standard of 50%. Additionally, while 74% of patients were seen by a dermatologist within the two-week timeframe, this did not meet the 100% standard set by NICE guidelines.

These findings not only demonstrate the exponentially increasing burden placed on tertiary specialist services but also highlight the need for improved referral efficiency and diagnostic accuracy within primary care. The contributing factors identified include limited post-graduate dermatology training for GPs and the promotion of referrals for uncertain lesions. Proposed interventions to enhance referral pathways include the development of e-learning modules to improve GP education, the implementation of teledermatology and artificial intelligence services to effectively triage cases, and regular reviews of referral patterns by local primary care services. Through earlier and more targeted diagnosis, the suggested techniques may improve the effectiveness of the referral process and have the potential to improve outcomes for patients with suspected skin malignancies.

## Introduction

Skin cancers are among the most prevalent cancers in the Western world, of which there are three broad subtypes: squamous cell carcinomas (SCC), basal cell carcinomas, and melanomas. Their incidence rates have risen significantly, with cutaneous melanomas increasing annually by 3-7% among the Caucasian population over the past two decades [1]. In the UK, skin malignancies are becoming increasingly frequent; in 2016, melanomas were identified as the fifth most common malignancy, affecting up to 16,000 patients [2]. Furthermore, a systematic study by Lomas et al. in 2012 highlighted that the incidence of keratinocyte carcinomas had increased by 61% in the last ten years, and the incidence of non-melanotic skin cancer in the UK was increasing at a faster rate than in other European nations [3]. The COVID-19 pandemic exacerbated the issue of reduced diagnostic accuracy (i.e., reduced confirmed and delayed diagnoses), leading to a 68.1% reduction in positive diagnoses [4] and significant delays in assessing referrals. This potentially could be attributed to the reluctance of patients to seek medical attention [5]. 

The pathway of diagnosing skin cancers remains multifaceted; however, it is evident that primary care plays a vital role in this process, as they remain the initial healthcare professionals who interact with patients prior to referral. Within general practitioner (GP) consultations, these professionals are tasked with distinguishing between malignant and benign lesions. Suspected lesions are then referred under the two-week wait pathway for further specialist assessment. The importance of early detection of such cancers cannot be overstated; for instance, early melanoma diagnosis results in a 95% five-year survival rate [2].

Whilst this pathway has been effective in identifying skin cancers, it is evident from the literature that only a small proportion of referrals result in a confirmed diagnosis [2], underscoring the challenges faced in primary care. Addressing these challenges could prove highly beneficial in reducing unnecessary dermatology referrals, alleviating the burden placed on tertiary specialist services, and improving diagnostic accuracy for skin cancers.

Aims

The primary outcome of this audit was to identify the number of fast-track referrals for suspected skin cancers and to determine the proportion of referrals resulting in a positive diagnosis of skin cancer, including squamous/basal cell carcinomas and melanomas. Through these findings, this audit’s goal was to evaluate potential reasons for positive/negative diagnoses of skin cancers in primary care, alongside potential solutions to reduce the number of referrals resulting in a negative diagnosis and improve primary care screening of skin cancers. Additionally, we aimed to review whether the referral appointments were seen within two weeks by a dermatologist as per National Institute for Health and Care Excellence (NICE) guidelines.

## Materials and methods

This single-centre retrospective clinical audit was conducted at Castle Healthcare Practice, a GP practice based in West Bridgford, Nottingham, canvassing approximately 17,000 patients.

This audit adhered to the guideline established by the National Institute for Health and Care Excellence (NICE) [6], whereby the following standard was set with regard to fast-track referrals for suspected skin cancers. Standard 1 included 0% of fast-track referrals not to be seen by dermatology within two weeks. 

In addition to the standard set by NICE, we used a local audit benchmark set by the GP partners at Castle Healthcare Practice in order to reflect the desired specificity in referral practices. Standard 2 included 50% of fast-track referrals to dermatology, resulting in a positive diagnosis for skin cancer, including squamous/basal cell carcinoma and melanomas.

Eligibility criteria 

The eligibility criteria for which referrals were selected were as follows: in the time period between 8th September 2021 and 28th July 2022, in order to ascertain an appropriate number of referrals to analyse and assess significant trends; referral letters under the fast track two-week wait referral pathway, excluding dermatology referrals that were referred under longer pathways, i.e., four to six weeks for specialist review rather than urgent skin carcinoma assessments.

Search strategy 

We conducted a comprehensive search of the electronic database known as *SystmOne*, an electronic health records system used widely across NHS practices, using the following search strategy: (“Basal Cell Carcinoma” OR “BCC” OR “Squamous Cell Carcinoma” OR “SCC” OR “Melanoma”) AND “Skin Cancer.” These keywords were used in order to accurately identify confirmed skin cancer diagnoses. 

Data collection and analysis

Through this strategy, a total of 146 referral letters were identified, encompassing all age groups and demographics. We then organised an electronic spreadsheet to collect the data in a systematic manner, removing direct patient identifiers, including NHS numbers and addresses, in order to remove any unwanted bias. For our primary outcome, we recorded the number of these referrals that resulted in a confirmed, i.e., positive diagnosis of skin cancer by reviewing each patient’s clinic letter by a dermatologist within their records looking for the keywords mentioned above.

For our secondary outcome, the date of the dermatology appointment post-referral was noted by reviewing the tabbed journal and referral letter sections in *SystmOne*. This allowed a comparison of the initial referral date with the date of the dermatology appointment to evaluate whether the two-week referral guidelines were fulfilled. However, due to unforeseen time constraints, we were unable to analyse all 146 referral letters for this secondary outcome. Hence, in order to obtain a representative sample from which we could extrapolate the data, we used a stratified random sample approach, splitting all the referrals into groups according to age groups, and through a random number generator, selecting a total of 35 referrals for further analysis in order to be as representative of the sample. 

## Results

**Figure 1:**
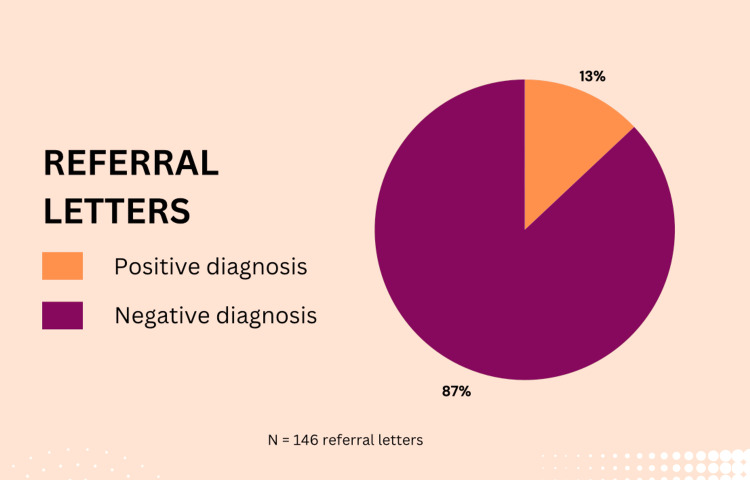
The proportion of referral letters between September 8, 2021, to July 28, 2022 with n=146 (number of two-week wait referrals to dermatology). Positive diagnosis means a confirmed skin cancer diagnosis (squamous cell carcinomas, basal cell carcinoma, or melanoma)

**Figure 2:**
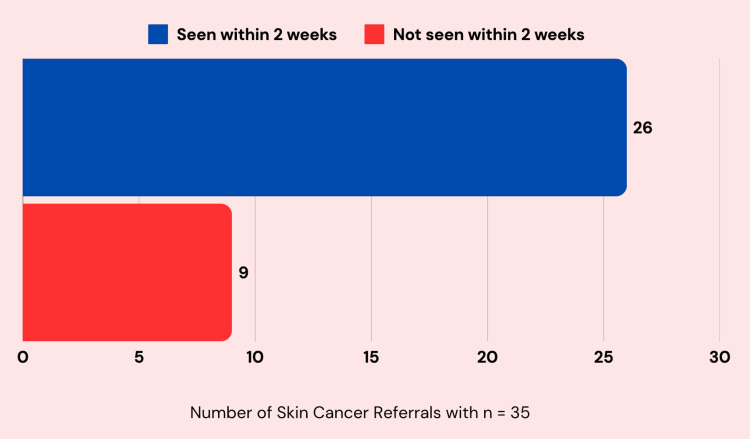
The proportion of fast-track referrals in a sample subset (n=35) that were seen within two weeks.

Of the referral letters identified between September 8, 2021, and July 28, 2022, only 19 (13%) resulted in a confirmed diagnosis of skin cancer (Figure 1). This proportion fell significantly short of the standard set for this study, which aimed for 50% of referrals leading to a diagnosis of skin cancer. NICE guidelines [6] stipulate that patients referred under the fast track pathway should be seen within a maximum of 14 days. In order to quantify compliance with this guideline, a subset of 35 patients was randomly selected. Twenty-six patients (74%) adhered to this timeframe, whereas nine patients (26%) experienced delays beyond two weeks (Figure 2). In those experiencing delays, the waiting interval ranged from 15 to the longest delay observed at 35 days. While the 74% compliance rate indicates that a majority of patients received timely care, it still falls short of the 100% target outlined in the study’s criteria. These findings underscore the need to address issues that may contribute to delays in accessing specialist dermatology services.

## Discussion

Within the UK, the fast-track referral pathway has proven essential in detecting and referring suspected skin malignancies to tertiary specialised services to prompt treatment. However, as demonstrated by this audit’s findings, the low diagnostic accuracy of this pathway has posed potential drawbacks to this approach. This issue is a recurring theme within current literature [7], with similar studies performed by Westbrook et al. (2006) showing a 19.5% specificity [8] and Shariff et al. (2007) showing a 34.5% specificity in their sample size [9]. 

Analysis of the 19 confirmed cases in this study revealed common high-risk factors, including a family history of skin cancer, significant sun exposure, rapid lesion growth, and irregular lesion borders. Of note, SCC and melanoma cases accounted for six of the confirmed diagnoses, underscoring the particular challenges associated with identifying these more aggressive cancers. Our findings point to the necessity of increasing awareness of screening tools in order to aid primary care physicians in distinguishing between malignant and benign lesions. An example of a screening tool used more recently in primary care is the Glasgow seven-point checklist (Table 1) [9,10]. The implementation and increased awareness of this tool has shown a degree of success in improving specificity in skin cancer referrals, with Walter FM et al. in 2013 [11] demonstrating an increase in positive predictive value (PPV) of 5.3% post-increasing the revision in the cutoff score to 4 from 3. This change in the cut-off score of this diagnostic tool, in conjunction with the well-known ABCDE technique (Table 2), could possibly offer solutions to the diagnostic accuracy. 

**Table 1:** The Glasgow seven-point checklist lesion system seen in the Scottish Intercollegiate Guidelines Network (SIGN) in 2023. A score of >3 is generally accepted as a cutoff for suspicion of skin cancer. [10]

Major Features (2 points)	Minor Features (1 point)
Change in size of lesion	Inflammation
Irregular pigmentation	Itch/altered sensation
Irregular border	Lesion larger than others
	Oozing/crusting of lesion

**Table 2:** The more well known ABCDE approach used to assess suspicious skin lesions, seen in Scottish Intercollegiate Guidelines Network (SIGN). [10]

A	Geometrical Asymmetry in 2 Axes
B	Irregular border
C	At least 2 different colours in lesion
D	Maximum diameter >6mm
E	Evolution/change in lesion

The causative factors contributing to the low diagnostic accuracy observed within this and other studies are multifactorial. One recurring theme amongst these studies is the limited post-graduate dermatology training provided to primary care physicians. In the UK, postgraduate training in dermatology is not compulsory and is limited to a select group of primary care physicians who choose dermatology as a hospital rotation during their four-month placement. It is also briefly covered in the Membership of the Royal College of General Practitioners (MRCGP) (GP speciality) examinations as part of the curriculum. However, it is clear from the literature that primary care physicians view dermatology as an understated and under-taught part of their curriculum, with one survey carried out by Kerr et al. [12] highlighting that 71% of GPs believe that dermatology should be taught at the postgraduate level and is of equal importance as other specialities that are considered compulsory within the GP’s training pathway, such as acute medicine and paediatrics.

This is further strengthened by additional studies, such as Göl İ et al. in 2018 [13], identifying that 38% of GPs lacked sufficient knowledge to perform adequate skin lesion assessments, including the screening tools mentioned earlier in this article. Therefore, the use of educational interventions to aid primary care physicians in their decision-making when assessing suspicious skin lesions could prove vital; for instance, the use of e-learning modules raising awareness of screening tools, live teaching sessions, and online courses. Brown et al.’s [14] systematic review found that interactive e-learning modules improved understanding of skin cancer risk factors and symptoms. However, the impact of such interventions on clinical practice remains uncertain.

Teledermatology offers an encouraging solution to address diagnostic challenges and reduce unnecessary referrals. This approach involves sharing digital images of skin lesions with dermatology specialists through a rapid online service, enabling more accurate triage and diagnosis [15]. A UK study demonstrated that incorporating tele-dermatoscopy into primary care reduced referrals by 58% [16]. 

While technological advancements in skin cancer assessment will play a crucial role in early detection, it is important to note that not all interventions may prove successful. Upon examination of the literature, the use of smartphone applications has been trailed as an approach to identify pigmented lesions earlier using a smartphone camera and underlying algorithm within the application, to low levels of success. For instance, Ngoo et al. [17] and Freeman et al. [18] both demonstrated that currently, the algorithm-based smartphone applications were associated with a high likelihood of missing melanomas and low diagnostic accuracy in comparison to a dermatologist evaluation. Whilst this data is not particularly encouraging, it is important to note that these applications are in the early stages of clinical application and do require further assessment/development alongside current management guidelines to provide more value.

The ever-growing influence of artificial intelligence within healthcare remains a controversial topic that could further play a role in aiding diagnostic accuracy in primary care. Recent literature attributed to the success of AI-based classification of skin cancers in comparison to dermatologists is conflicting, with select comparative studies [19,20] showing a higher specificity for both AI systems and dermatologists when pitted against each other. However, more importantly, a combination of both AI-based and specialist dermatologist input into primary care services may achieve the highest success rates in accurately diagnosing skin cancers [21].

While the cost-effectiveness and feasibility of technological interventions such as teledermatology, smartphone applications, and AI-based classification require further evaluation, there is significant potential to alleviate pressure on dermatology services.

Recommendations 

The primary issues highlighted within the skin cancer referral pathway included educational and technological limitations in the general practice. Improving education regarding the accurate diagnosis of skin cancers is an important step in lowering the incorrect referral rate, as greater confidence amongst general practitioners of all levels would reduce the number of benign lesions being sent to secondary care. The production of an e-learning tool, such as a teaching module, may improve the education of clinicians by promoting greater awareness of risk factors for skin cancer, showcasing their manifestations on a range of different skin types, and highlighting patient-guided self-examination techniques for safety netting. An alert system for two-week wait referrals from primary care would help to ensure rapid follow-ups if appointment letters did not reach patients within four weeks. Furthermore, biannual reviews of the referral patterns within each practice could highlight previously unidentified issues resulting in unnecessary referrals, thus maintaining a more accurate system. In addition, expanding teledermatology services to GP practices may allow for faster access to specialists when considering borderline lesions. This not only will allow more experienced clinicians to offer advice for more difficult cases but could reduce the duration of primary care consultations and their workload. Finally, it is imperative that re-auditing of the adherence to these standards is performed on an annual basis in order to highlight the impact of these interventions on referral accuracy and to identify any further gaps.

Limitations 

The main limitation of this study is related to its small sample size, as 23.3% (n=146) letters were analysed with regard to compliance with two-week wait guidelines and positive diagnoses. The data was collected from a single GP practice, which may limit the generalisation of the findings and reduce external validity, and a stratified sample size was collected, which may not be truly representative of the sample. Additionally, discrepancies in clinical coding due to human error within the *SystmOne* database may have led to incomplete identification of referrals with confirmed diagnoses of skin cancer.

In addition, the use of a 50% standard for diagnostic confirmation (standard 2) is specific to this audit as it is a locally set benchmark and may not be directly comparable to national guidelines such as the NICE recommended positive predictive value (PPV) threshold of 3%. The purpose of the 50% standard was to represent an aspirational target for optimising diagnostic specificity within the context of the local general practice. While the NICE PPV threshold establishes a minimal level for referral in order to maintain sensitivity, this audit aimed to assess and enhance the balance between sensitivity and specificity, with the goal of reducing unnecessary referrals without compromising the detection of genuine cases.

## Conclusions

Overall, this retrospective clinical audit identified a number of key factors limiting the efficacy of the referral pathway for suspected skin cancers in a GP setting. While a 74% compliance with the two-week wait NICE standard was elicited, the overall diagnostic accuracy was still well below the expected standard of 50%. As a result, a range of targeted approaches with the aim of improving the diagnosis of dermatological malignancies is crucial in early identification and improving patient outcomes. The combination of teaching programs with modern educational methods alongside technological interventions such as teledermatology/artificial intelligence may help to improve diagnostic precision and streamline the process of skin cancer referrals.
